# Disentangling Social Determinants of Health and Rurality in Head and Neck Cancer 2‐Year Mortality

**DOI:** 10.1002/oto2.62

**Published:** 2023-07-07

**Authors:** Celina Virgen, Bryan Renslo, Tuleen Sawaf, Yelizaveta Shnayder, Kiran Kakarala, Andrés M. Bur, Kevin J. Sykes

**Affiliations:** ^1^ Department of Otolaryngology–Head and Neck Surgery University of Kansas Medical Center Kansas City Kansas USA

**Keywords:** head and neck cancer, health disparities, rural, social determinants of health

## Abstract

Social determinants of health (SDoH) and rurality are known factors that may influence outcomes in head and neck squamous cell carcinoma (HNSCC). Patients residing in remote locations or those with multiple SDoH may encounter barriers to initial diagnosis, adherence to multidisciplinary treatments, and posttreatment surveillance, which may impact their overall survival. However, previous studies have shown mixed results associated with rural residence. The aim of this study is to identify the impact of rurality and SDoH on 2‐year survival in HNSCC. The study was conducted using a Head and Neck Cancer Registry at a single institution from June 2018 through July 2022. Rurality, defined by US census scores, and individual measures of SDoH were used. Our results indicate that each additional adverse SDoH factor results in 1.5 times the odds of mortality at 2 years. Individualized measures of SDoH, rather than rurality alone, better reflect patient prognosis in HNSCC.

Care for head and neck squamous cell carcinoma (HNSCC) often requires a multidisciplinary approach from initial presentation through surveillance. Disparities in recent survival trends likely reflect differences in access and adherence to care, which are often rooted in inequities at the individual, community, and society levels. To date, there are limited studies evaluating these socioecological factors in individual HNSCC patients.

Social determinants of health (SDoH) are conditions that influence health outcomes such as education, economic stability, and community.^1^ Adverse SDoH disadvantage patients from accessing health care and are associated with decreased life expectancy.^2^ Studies assessing the influence of rurality on HNSCC outcomes have shown varying results.^3^, ^4^, ^5^, ^6^ Given these findings, the aim of this study is to distinguish the influence of SDoH and rurality on 2‐year survival in HNSCC patients.

## Methods

Data were prospectively collected through a Head and Neck Cancer Registry (HNCR) at the University of Kansas Medical Center designed to evaluate the influence of SDoH on clinical and quality outcomes. Participants were consented and enrolled from June 2018 to July 2022 under institutional review board approved protocol (#00001732). The data were retrospectively analyzed to identify patients with 2‐year data. Inclusion criteria consisted of adult patients, biopsy‐positive squamous cell carcinoma of the oral cavity, oropharynx, or larynx. Human papillomavirus (HPV)‐related cancers were excluded due to better prognosis than HPV‐negative cancers.^7^, ^8^

To identify levels of SDoH, each demographic factor was categorized as individual (Level 1) or community (Level 2) factors (Table 1). Measures of SDoH were employment, education, health literacy, and insurance status. All data were collected at the time of enrollment into the HNCR. Neighborhood socioeconomic status was measured using the area deprivation index (ADI). Adverse contributions from SDoH were categorized based on previous literature investigating the impact of each factor on patient outcomes.^9^, ^10^, ^11^, ^12^, ^13^, ^14^, ^15^, ^16^, ^17^ SDoH were scored based on a protective or adverse classification. Higher total SDoH scores reflected a greater sum of adverse SDoH factors when analyzing cumulative effect. The 2013 Rural‐Urban Continuum Code (RUCC) from the US Department of Agriculture was used to code residence.^18^ RUCC classification of 1 or 2 is considered urban while 3 to 9 are rural. Addresses were accessed from the electronic medical record and geocoded to latitude and longitude coordinates through Geoapify28.^19^ This was combined with the corresponding polygon based on their census block using ArcGIS Pro (Esri, Version 2.9). Only residents from Kansas or Missouri were included. PO box address was provided by four participants for which ADI was based on location.

**Table 1:** Social Determinants of Health Scale

		0 points if…	1 point if…
Level 1 individual	Employment	Employed (full‐time or part‐time)	No employment or retired
	Education level	College graduate	Less than college education
	Health literacy	Adequate level	Less than adequate
	Insurance status	Private or medicare	Medicaid or no insurance
Level 2 environment	ADI (national)	Greater than 59.3	Less than 59.3
	Total = 0–5^a^		

Abbreviations: ADI, area deprivation index; SDoH, social determinants of health.

^a^ Higher score indicates a higher degree of negative SDoH.

Data analysis includes Pearson's *χ* ^2^ test, Fisher's exact test, and Wilcoxon rank‐sum test for continuous or categorical variables. Univariate logistic regression was performed to generate crude odds ratios (OR). Stepwise multivariable analysis was utilized to find the model of best fit, and adjusted ORs were generated. An *α* threshold of *α* = .05 was applied for significance a priori. All statistical testing was performed using RStudio version 4.2.1.

## Results

A total of 161 patients were included with 136 alive (Group 1) and 25 deceased (Group 2) at 24 months posttreatment. The mean age of patients was 61.8 years (SD: 11.6). A higher proportion of patients in Group 2 had Stage IV disease (n = 18, 72%) compared to Group 1 (n = 60, 46.2%). There were no significant differences between the groups in age, gender, rurality, employment, education, health literacy, insurance, or community (Table 2).

**Table 2:** Patient Demographics and Characteristics

Characteristic	Overall, no. (%)	Alive, no. (%)	95% CI	Deaths, no. (%)	95% CI	*p* value
n	161	136		25		
Rurality (RUCC)	69 (42.9)	59 (43.4)	35.0, 52.1	10 (40.0)	21.8, 61.1	.753
Site						
Oral cavity	120 (74.5)	104 (76.5)	68.3, 83.1	16 (64.0)	42.6, 81.3	.126
Oropharynx	15 (9.3)	10 (7.4)	3.78, 13.5	5 (20.0)	7.61, 41.3	
Larynx	26 (16.1)	22 (16.2)	10.6, 23.7	4 (16.0)	5.25, 36.9	
Age, mean (SD)	61.8 (11.6)	61.2 (11.6)	59.2, 63.2	64.8 (11.1)	60.3, 69.4	.075
Stage						.157
I	40 (24.8)	36 (26.5)	19.5, 34.8	4 (16.0)	5.25, 36.9	
II	17 (10.6)	15 (11.0)	6.51, 17.8	2 (8.0)	1.40, 27.5	
III	22 (13.7)	21 (15.4)	10.0, 22.9	1 (4.0)	0.21, 22.3	
IV	82 (50.9)	64 (47.1)	38.5, 55.8	18 (72.0)	50.4, 87.1	
Female	60 (37.3)	51 (37.5)	29.5, 46.2	9 (36.0)	18.7, 57.4	.887
Employment	89 (55.3)	72 (52.9)	44.2, 61.5	17 (68.0)	46.4, 84.3	.164
ADI	78 (48.4)	64 (47.1)	38.5, 55.8	14 (56.0)	35.3, 75.0	.411
Education	81 (50.3)	64 (47.1)	38.5, 55.8	17 (68.0)	46.4, 84.3	.054
Health literacy	109 (67.7)	90 (66.2)	57.5, 73.9	19 (76.0)	54.5, 89.8	.334
Insurance	10 (6.2)	8 (5.9)	2.76, 11.6	2 (8.0)	1.40, 27.5	.655
Overall SDoH						.142
0	10 (6.2)	10 (7.4)	3.78, 13.5	0 (0.0)	0, 16.6	
1	34 (21.1)	31 (22.8)	16.2, 30.9	3 (12.0)	3.15, 32.3	
2	45 (28.0)	37 (27.2)	20.1, 35.6	8 (32.0)	15.7, 53.6	
3	46 (28.6)	39 (28.7)	21.4, 37.2	7 (28.0)	12.9, 49.6	
4	25 (15.5)	19 (14.0)	8.83, 21.2	6 (24.0)	10.2, 45.5	
5	1 (0.6)	0 (0.0)	0, 3.42	1 (4.0)	0.21, 22.3	
Overall SDoH (continuous)	2.3 (1.2)	2.2 (1.2)	2.0, 2.4	2.8 (1.1)	2.3, 3.2	.039

Abbreviations: ADI, area deprivation index; CI, confidence interval; RUCC, Rural‐Urban Continuum Codes; SD, standard deviation; SDoH, social determinants of health.

When assessing factors influencing survival, rurality was not significantly associated (OR: 0.87; 95% confidence interval [CI]: 0.36, 2.06; *p* = .75). Likewise, individual measures of SDoH did not significantly influence survival. By contrast, a higher total SDoH score was significantly associated with decreased overall survival on univariate (*p* = .039) and multivariable analysis (OR: 1.57; 95% CI: 1.07, 2.38; *p* = .027) (Table 3). All other variables were removed from multivariate analysis due to statistical insignificance.

**Table 3:** Patient Characteristics, Univariate and Multivariate Analysis (N = 161)

	Univariate		Multivariable	
Characteristic	OR (95% CI)	*p* value	aOR (95% CI)	*p* value
Age	1.03 (0.99–1.07)	.152		
Site				
Oral cavity	—			
Oropharynx	3.25 (0.92–10.5)	.053		
Larynx	1.18 (0.32–3.60)	.783		
Stage				
I	—			
II	1.20 (0.15–6.86)	.843		
III	0.43 (0.02–3.14)	.462		
IV	2.53 (0.86–9.27)	.116		
Rurality (RUCC)	0.87 (0.36–2.06)	.754		
SDoH factors				
Employment	1.89 (0.78–4.90)	.169		
Education level	2.39 (0.99–0.20)	.059		
Health literacy	1.62 (0.63–4.69)	.338		
Health insurance	1.39 (0.20–6.00)	.688		
ADI	1.43 (0.61–3.44)	.413		
SDoH scale	1.57 (1.07–2.38)	.027	1.57 (1.07–2.38)	.027^a^

Abbreviations: ADI, area deprivation index; aOR, adjusted odds ratio; CI, confidence interval; OR, odds ratio; RUCC, Rural‐Urban Continuum Codes; SDoH, social determinants of health.

^a^ Multivariate analysis (all other variables not included in the model due to statistical insignificance).

## Discussion

The current study investigated the influence of adverse SDoH on survival, distinguishing these factors from rurality. This distinction is critical when applying results from national database studies to individual patients in practice. The present study found no significant differences in overall survival between rural and urban residents. While there are mixed results between survival and rurality, our findings align with studies where no significant differences in survival were found.^3^, ^4^ This suggests that patients with positive SDoH residing in remote communities are able to access timely high‐quality therapy. Therefore, a better understanding of patients' social‐ecological context may inform personalized risk stratification and care pathways.

The cumulative effect of multiple adverse SDoH predicted decreased survival. Similarly, Pinheiro et al^20^ studied the effect of SDoH on all cancer mortality using a longitudinal prospective database. The authors determined a greater number of SDoH was associated with increased cancer mortality risk, controlling for all other factors. This negative effect has also been observed in other disciplines with chronic kidney disease and incidence of stroke.^21^, ^22^

Findings from our study demonstrate the impact of adverse SDoH on HNSCC outcomes. As this study was limited into a single center, these findings warrant further investigation in additional prospective datasets. Future studies may use these personalized measures of SDoH to develop a more robust scale of risk factors. Early targeted referrals based on these factors could improve prognosis for these patients.

## Conclusion

The cumulative effect of adverse SDoH has a stronger influence on 2‐year survival in HNSCC than rurality alone. The results from this study highlight the need for more individual measures of SDoH in the consideration of HNSCC survival. Consistent and personalized screening for SDoH is needed to better understand their influence on prognosis.

## Author Contributions

**Celina Virgen**, **Bryan Renslo**, and **Tuleen Sawaf**, study design, data collection, data analysis, and manuscript preparation; **Yelizaveta Shnayder**, **Kiran Kakarala**, **Andrés M. Bur**, and **Kevin J. Sykes**, study design and manuscript revisions.

## Disclosures

### Competing interests

None.

### Funding source

None.
